# Modulated Luminescence of Lanthanide Materials by Local Surface Plasmon Resonance Effect

**DOI:** 10.3390/nano11041037

**Published:** 2021-04-19

**Authors:** Jinhua Liu, Qingru Wang, Xu Sang, Huimin Hu, Shuhong Li, Dong Zhang, Cailong Liu, Qinglin Wang, Bingyuan Zhang, Wenjun Wang, Feng Song

**Affiliations:** 1 School of Physical Science and Information Technology, Shandong Provinical Key Laboratory of Optical Communication Science and Technology, Liaocheng University, Liaocheng 252059, China; ljh794982331@126.com (J.L.); lishuhong@lcu.edu.cn (S.L.); zhangdong@lcu.edu.cn (D.Z.); cailong_liu@jlu.edu.cn (C.L.); wangqinglin@lcu.edu.cn (Q.W.); zhangbingyuan@lcu.edu.cn (B.Z.); wjwang@lcu.edu.cn (W.W.); 2 School of Physics, Nankai University, Tianjin 300071, China; 2120180202@mail.nankai.edu.cn (X.S.); 2120170177@mail.nankai.edu.cn (H.H.)

## Abstract

Lanthanide materials have great applications in optical communication, biological fluorescence imaging, laser, and so on, due to their narrow emission bandwidths, large Stokes’ shifts, long emission lifetimes, and excellent photo-stability. However, the photon absorption cross-section of lanthanide ions is generally small, and the luminescence efficiency is relatively low. The effective improvement of the lanthanide-doped materials has been a challenge in the implementation of many applications. The local surface plasmon resonance (LSPR) effect of plasmonic nanoparticles (NPs) can improve the luminescence in different aspects: excitation enhancement induced by enhanced local field, emission enhancement induced by increased radiative decay, and quenching induced by increased non-radiative decay. In addition, plasmonic NPs can also regulate the energy transfer between two close lanthanide ions. In this review, the properties of the nanocomposite systems of lanthanide material and plasmonic NPs are presented, respectively. The mechanism of lanthanide materials regulated by plasmonic NPs and the scientific and technological discoveries of the luminescence technology are elaborated. Due to the large gap between the reported enhancement and the theoretical enhancement, some new strategies applied in lanthanide materials and related development in the plasmonic enhancing luminescence are presented.

## 1. Introduction

The lanthanide elements in the periodic table belong to the same group of IIIB. These elements have the unique 4f electronic structures. Their long emission lifetimes, narrow emission bandwidths, and excellent photo-stability have attracted much attention due to their potential applications in laser [1], Light emitting diode(LED) [2], biological applications [3], and solar cells [4,5]. However, the challenge of lanthanide materials is the relatively low luminescence efficiency due to the small photon absorption cross-section, the complex energy level structure of lanthanide ions (Ln^3+^), and many non-radiative processes in a certain matrix environment. Improving the luminescence efficiency and regulating the luminescence properties of lanthanide materials have become the focus on the research of these materials. Many ways to enhance luminescence have been demonstrated, such as high-temperature solid status reaction method [6], electric field enhancement [7], aggregation-induced emission [8], and so on. Remarkable progress has been made over the past ten years in lanthanide materials’ luminescence enhancement, which leads to the modification of the excitation or emission process and the alterations of luminescence lifetimes and quantum yields of lanthanide materials. Among them, the localized surface plasmon resonance (LSPR) effect of nanoparticles (NPs) has been developed as a valuable strategy [9,10,11,12]. The interaction of light with noble metal NPs produces a collective oscillation of conduction band electron known as LSPR. Here, we focus on the luminescence of lanthanide materials influenced by plasmonic nanomaterials based on the LSPR effect. In recent years, the noble metal NPs have been promising plasmonic NPs used for enhancing and modulating the luminescence of lanthanide materials. The enhanced and modulated luminescence is usually associated with the effect of LSPR, which gives rise to strong, local electromagnetic field enhancement or radiation decay rate increase [9,13]. Coupling the lanthanide elements with the LSPR effect of plasmonic nanomaterials to modulate the luminescence efficiency has become an improvement strategy by designing and tuning the structure, size, and shape of the plasmonic nanomaterials.

**Figure 1:**
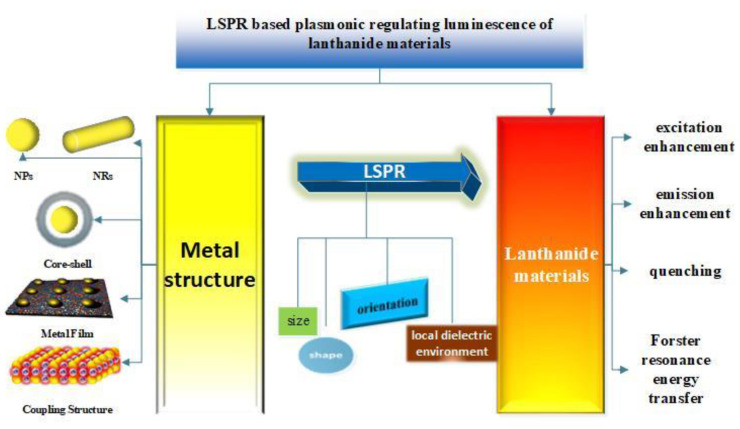
Table of contents (TOC) drawing of localized surface plasmon resonance (LSPR)-based plasmonic regulating luminescence of lanthanide materials. Image of metal film and coupling structure, from Reference [14] and Reference [12].

In this work, we offer a general overview of the interactions between plasmonic NPs and lanthanide ion-doped luminescent materials, which provide a great deal of new properties and novel applications for lanthanide materials. The paper is arranged as follows. Figure 1 shows the schematic of LSPR-based plasmonic regulating luminescence of lanthanide materials. In Section 2, we will start with a brief introduction of the basic principles underlying the LSPR effect and the luminescence mechanism of lanthanide materials respectively, followed by a discussion on the luminescence of materials influenced by noble metal NPs in the four relative aspects: the excitation enhancement induced by enhanced local electric field, emission enhancement induced by increased radiative decay rate, quenching induced by energy transfer from lanthanide ions to plasmonic NPs, and the enhanced luminescence by energy transfer from plasmonic NPs to lanthanide ions, in Section 3. Finally, the new thinking and methods applied in plasmonic enhancing luminescence and the potential applications, and future possible directions of LSPR in the lanthanide materials, will be discussed in Section 4. Section 5 concludes the paper.

## 2. Theory for LSPR and Lanthanide Materials

### 2.1. LSPR Effect

Metal contains the stationary positive ions and high-speed movement of free electrons. The free electrons can be regarded as no interaction of ideal gas. To remain electrically neutral, it is assumed that the positive charge of the positive ion is distributed throughout the volume, neutralizing the negative charge of the free electron. This model is like a conventional plasma, and therefore it is called a plasma in a metal. The plasmon is a collective oscillation of the free electrons in a noble metal. This free electron response is described by the dielectric function of the metal as per the Drude model: $\varepsilon_{Drude}=1-\frac{\omega_{p}^{2}}{\omega^{2}+i\gamma \omega }$, where ω is the angular frequency of the light, γ is the damping parameter in the bulk, and $\omega_{p}$ is the bulk plasma frequency of the free electrons, which is determined by the density of free electrons, N, in the metal and the effective mass, $m_{e},$ of the electrons, as: $\omega_{p}^{2}=\frac{Ne^{2}}{\varepsilon_{0}m_{e}}$, where $\varepsilon_{0}$ is the permittivity of free space and e is the electron charge. For visible and near infrared frequencies, ω, γ≪ω, so the above can be simplified to $\varepsilon_{Drude}=1-\frac{\omega_{p}^{2}}{\omega^{2}}$ [15,16,17].

Plasmons resonate in the form of surface plasmon polaritons (SPPs) at the surface of a metal, which is named surface plasmon. Only materials with a negative real and small positive imaginary dielectric constant are capable of supporting surface plasmon. The most common materials used are Au and Ag. If the surface plasmon is confined to a particle, which size is comparable to the wavelength of light, it is localized surface plasmon (LSP). When the incident electromagnetic field matches that of the oscillating electrons on the surface of plasmonic NPs, a resonance condition is met, which produces a collective oscillation of a conduction band electron known as LSPR. At this moment, the real part of the noble metal dielectric function satisfies equation: $Re\left(\varepsilon \right)=-\kappa \varepsilon_{m}$, here Re denotes the real part, κ is a shape factor that incorporates the dependence of the polarizability on the geometry of the surface that defines the electron oscillations, and $\varepsilon_{m}$ is the medium dielectric constant [17,18]. This is a non-propagating form of plasma oscillation compared to the conduction surface plasmon, and it is localized on the surface of NPs or discontinuous nanostructures. In the meantime, the LSPR produces large, wavelength-selective increases in absorption, scattering, and electromagnetic field at the nanoparticle surface. Usually, the enhancement of the localized electromagnetic field is called the local field effect. Now, the LSPR effect in the visible or infrared region has become the focus of research due to their ability to modulate the optical properties of luminescent materials [15,19].

When the LSPR effect is generated, it will be manifested as the strong extinction cross-section (scattering and absorption) of plasmonic NPs, which depends on the nanoparticles’ composition, size, shape, orientation, local dielectric environment, and the peak wavelength, $\lambda_{max}$, in particular [20]. The other effect is the greatly enhanced local electric field, which is strongest at the surface and quickly reduces with distance. Due to the unusual properties of the metal nanostructures, they are widely used in optical detection [21], molecular imaging [22,23], biosensors [24,25], and other fields [26,27,28]. Plasmonic NPs are also introduced into lanthanide luminescent materials, and the luminescent properties of lanthanide materials can be finely modulated by the LSPR effect.

### 2.2. Lanthanide Luminescent Materials

Lanthanide elements have a complex quasi-linear spectrum (narrow emission bandwidth) and unique character, different from those of transition metals, because of their unique 4f electronic structures. The outer 5s and 5p electrons shield the 4f electron shell. The location of lanthanide 4f and 5d levels within the band gap of compounds controls the lanthanide valence, luminescence, and charge carrier trapping properties. The f-f transitions strongly depend on the lanthanide ion, not the environment near the ions, which induced the narrow emission bandwidths. The Ln^3+^ have generous emission peaks in the visible and near infrared region. For instance, Tm^3+^ emits blue light, Sm^3+^ orange light, Tb^3+^ green light, Eu^3+^ red light, Yb^3+^, Nd^3+^, and Er^3+^ are famous for their near-infrared luminescence and up-conversion (UC) emission, and so on. 

The lanthanide luminescence materials generally consist of a host and activated Ln^3+^ doping ions. The lanthanide luminescence can be classed into two groups according to the mechanism of luminescence, down-conversion (DC), and up-conversion (UC) emission processes. The DC process is that the higher energy photons are excited and then emit the lower energy photons, which obeys Stokes’ law. In contrast, the UC process emits higher energy photons by exciting the lower photons, which can generate visible light from long-wavelength excitation of near infrared light [29]. The fundamental drawback of UC materials is the relatively low luminescence efficiency. Several kinds of inorganic compounds, such as oxide, oxysulfide, fluoride, phosphate, and vanadate, have been extensively used as host materials. There are also some organic compounds, such as polymethyl methacrylate (PMMA), polyvinyl alcohol (PVA), and so on, used as the host. The host can act as a host crystal to hold the Ln^3+^ ions and sensitize the luminescence of Ln^3+^ ions.

The significant parameters characterizing the emission of a lanthanide ion are the luminescence efficiency, η, and the luminescence lifetime, τ. η and τ of lanthanide luminescence satisfy the equations: $\eta =\frac{R}{R+K}$, $\tau =\frac{1}{R+K}$, here R is the radiative transition rate, and K is the non-radiative transition rate which performs all possible non-radiative decay processes. The photo absorption cross-section of Ln^3+^ ions is very small and there are many energy transfer processes, non-radiative decay, and cross-relaxation processes, which induce the low luminescence efficiency of Ln^3+^ ions. There are many ways to enhance the luminescence of lanthanide materials [30,31,32,33,34,35]. In recent years, many researchers have found that the LSPR effect of noble metal nanomaterials has the great potential to modulate the luminescence of lanthanide materials.

## 3. Plasmon-Modulated Luminescence of Lanthanide Materials

If plasmonic NPs are introduced into lanthanide luminescent materials, the luminescent properties of lanthanide materials would be modulated by the LSPR effect generated by plasmonic nanostructures. The optical properties (luminescence efficiency, chromaticity, lifetime, and so on) of lanthanide materials would be adjusted by changing the parameters of NPs, such as size, shape, and so on. The metal-enhanced/quenched luminescence is attributed to the different mechanisms, including the excitation enhancement due to the enhanced local field, the emission enhancement due to the increased radiative decay rate, and the quenching caused by the non-radiative energy transferring from the lanthanide materials to the plasmonic NPs. In addition, the plasmon nanostructures can also be used as the sensitizer to modulate the process of Forster resonant energy transfer (FRET). Generally, excitation and emission are considered as two independent processes occurring at different wavelengths. Accordingly, the total enhancement can be split into an excitation enhancement and an emission enhancement [36]. The enhanced brightness ($\gamma_{APP}$) can be expressed as $\gamma_{APP}=\gamma_{ex}\gamma_{em}\eta_{coll}\sigma$, where $\gamma_{ex}$ is the metal-induced excitation rate of the fluorophore at the excitation wavelength, $\gamma_{em}$ is the metal-induced emission rate of the fluorophore at the emission wavelength, $\eta_{coll}$ is the collection efficiency of the far-field light under the experimental conditions, and σ is a normalization factor [37]. For the systems with similar experiment conditions, the σ and $\eta_{coll}$ factors should be similar and thus can be approximately neglected. The $\gamma_{ex}$ and $\gamma_{em}$ factors hence become the dominant factors to enhance the fluorescence. Different kinds of plasmon nanostructures, such as Ag, Au NPs, islands, and nanorods, were used to modulate $\gamma_{ex}$ and $\gamma_{em}$. These factors affecting luminescence enhancement will be introduced in the following, respectively.

### 3.1. Excitation Enhancement

As is known, plasmonic NPs interact with nearby luminescent molecules through surface scattering and absorption. The scattering of incident light by plasmonic NPs induces the local electric field enhancement, forming ‘hot spots’ between particles. This localized electromagnetic field enhancement activates the nearby luminescent molecules, and more ions are excited to the excited state, which increases the excitation efficiency and thus enhances the luminescence [10]. The increase of excitation efficiency has great relationships with the spectral overlap of the LSPR band and the excitation spectrum, and the distance between the luminescent molecules and plasmonic NPs [38]. However, absorption of incident lights by plasmonic NPs leads to photothermal conversion, thus the local temperature near the luminescent molecules is increased due to the plasmonic NPs, resulting in quenching of luminescence [39]. This effect can be ignored in weak excitation regimes [9], and we also do not discuss it here.

**Figure 2:**
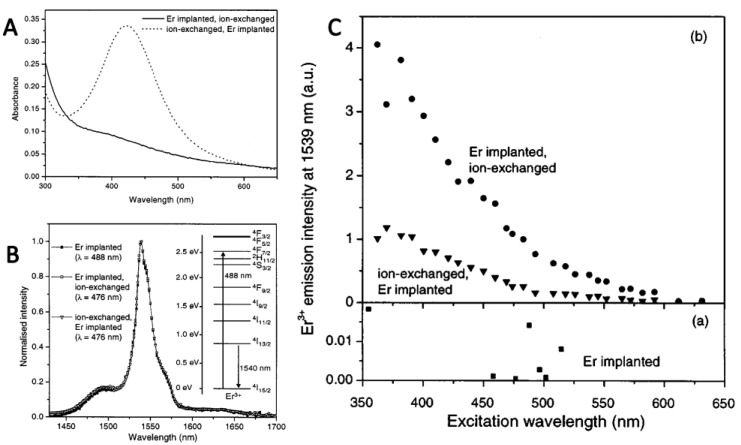
(**A**) Absorption spectrum of erbium-doped silver sample (ion-exchanged, Er implanted) and the reference is untreated glass (Er implanted, ion-exchanged). (**B**) Emission spectra of Er^3+^ material doped with Ag; the inset is image of the energy level scheme of Er^3+^. (**C**) Relationship between photoluminescence intensity and excitation wavelength of three different samples at 1539 nm: (**a**) Erbium-doped borosilicate glass (**b**). Erbium- and silver-doped borosilicate glass. Reference [41].

In the past decades, these ultrafine particles have been extensively studied by using different fabrication methods of glasses containing ultrafine Ag and Au particles. Hayakawa and his co-workers presented the excitation enhancement in the presence of silver particles of 4.3 nm size due to the local field enhancement [40]. Later, many researchers realized excitation enhancement by the enhancement of local field [41,42,43,44,45,46,47]. Figure 2 shows that the Er^3+^ excitation efficiency increased to 70 times when it was excited at 488 nm [41]. Meanwhile, researchers further found that the excitation enhancement also has a great relationship with the distance between the luminescent molecules and plasmonic NPs [38,48]. Moreover, the plasmon effect was applied in lanthanide ion-co-doped materials. Naczas reported an approach based on co-implantation of hydrogen and silver, which can improve PL of Er^3+^-doped silicon around 1540 nm. Both the excitation cross-section and the optical activation of Er^3+^ were increased [49]. The Ag NPs were nucleated inside the Tm^3+^, Eu^3+^, and Yb^3+^ triple doped system, and the up-conversion (UC) luminescence enhancement due to Ag NPs was investigated. After analysis, it was found that the enhancement was attributed to the local field increase due to the nearby Ag NPs but not due to the energy transfer from NPs to the lanthanide ions [50]. Recently, Saad and his co-worker indicated that the enhancement of the emission bands related to Eu^3+^ ions originated from the local field enhancement and the energy transfer from Ag NPs to Eu^3+^ ions, and from Dy^3+^ ions to Eu^3+^ ions [51]. These ultrafine Ag spherical particles exhibit a resonance at about 400 nm, which is generally matched with the excitation wavelength of many ultraviolet(UV)/blue excited lanthanide materials. Thus, the Ag spherical NPs with suitable size and space generally lead to local field enhancement.

**Figure 3:**
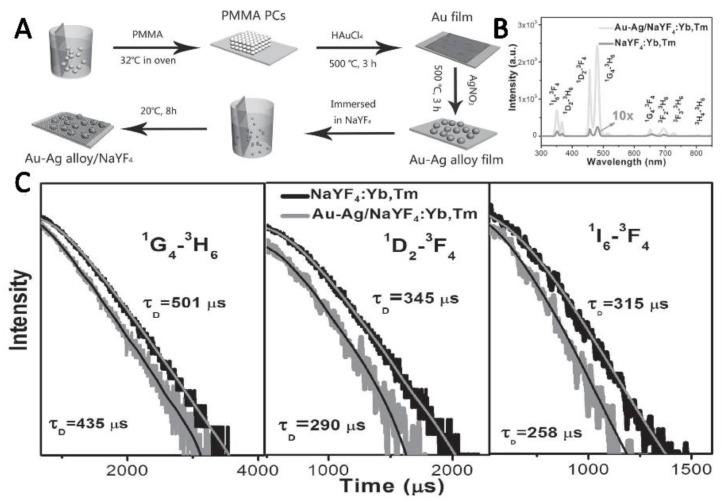
(**A**) Schematic diagram of the porous Au–Ag/NaYF_4_: Yb^3+^, Tm^3+^ composite film. (**B**) The UC luminescence(UCL) spectra of sample without and with Au–Ag films. (**C**) The UCL decay curves of different transitions when the samples were excited at 980 nm. Reference [53].

For the lanthanide-doped films, in order to enhance the local field, another approach is using NPs films or island films [52]. Chen prepared Au/Ag alloy film and obtained the large UC luminescence enhancement of Ln^3+^-doped NaYF_4_ composite films, where the enhancement factor can reach up to 180-fold. The enhancement was mainly attributed to the coupling of Au–Ag alloys and the excitation field of UC nanoparticle(UCNP) [53], as shown in Figure 3. Similar results were also obtained by using Au nanofilms with Ln^3+^-doped (β-NaYF_4_: Yb^3+^/Er^3+^) UCNPs [54]. The influence of distance between lanthanide-doped films and NPs films on luminescence enhancement was further performed by introducing space layer [55]. Kushlyk and his co-workers researched the Ce^3+^ and Yb^3+^ co-doped system with different NPs concentrations and sizes and proposed that plasmonic enhancement and quenching effect is a competition [56].

Another approach to enhance the local field is using the core shell nanostructures. The core shell nanostructures have the advantages of plasmonic NPs, and the distance between luminescent molecules and plasmonic NPs can be accurately controlled. One way is lanthanide NPs as the core or shell, and plasmonic NPs as the shell or core [57,58], this approach is generally applied in UC materials [59,60,61,62]. The other way is to add the prepared core-shell NPs into lanthanide materials. Som and Karmakar fabricated core-shell bimetallic NPs-embedded glass. The Au-Ag: Sm^3+^ co-embedded glass showed the fluorescence enhancement at 636 nm for Sm^3+^ ions. The enhancement originated from the local field enhancement [63]. Chu and his co-workers demonstrated the enhancement of lanthanides by Ag@SiO_2_ NPs. They found that the fluorescence enhancement mainly originated from the increase of the located electromagnetic field and the increase of the energy transfer [64,65]. Except the plasmonic NPs such as Au and Ag, other NPs which exhibit the LSPR effect were also used [66,67,68,69].

### 3.2. Emission Enhancement

The excitation enhancement generally increases the excitation rate but does not modify the emission processes, such as the luminescence efficiency, lifetime, and the radiative transition rate. When the LSPR band matches with the emission spectrum of lanthanide materials, the emission process can be modulated and its strength would be enhanced, and the luminescence efficiency and radiative decay rate would be greatly increased. The change in LSPR can be easily realized by tuning the shape, size, material, and local environment of NPs.

In the vicinity of plasmonic NPs, the excited molecules might emit photons directly to the far field or relax rapidly via nonradiative energy transfer to plasmonic NPs. The plasmonic NPs accept the energy and the LSPR is excited. The excited LSPR can emit luminescence at resonant wavelength in a radiative form or release the energy in a non-radiative form. If the LSPR matches with the emission spectrum of luminescent molecules, the LSPR inclines to take the radiative form and can result in the increase of radiative decay rate, which can also be explained by the Purcell effect [70]. According to the principle of the Purcell effect, the increase of radiative decay rate for lanthanide materials originates from the larger photon states’ density due to plasmonic NPs [71]. Plasmonic NPs can be regarded as a dedicated resonant cavity. Purcell suggested that the local photon states’ density would be obviously increased by placing molecules inside the plasmonic resonant cavity and thus the radiative decay rates of Luminescent molecules would be increased. The ratio of the increased and free-space emission rates can be described via the Purcell factor, $F=\frac{\gamma_{sp}}{\gamma_{0}}=\frac{3}{4\pi^{2}}(\frac{Q}{V_{\mathrm{mod}e}}){\left(\frac{\lambda }{n}\right)}^{3}$, where $\gamma_{sp}$ is the spontaneous emission rate of an emitter in a resonant cavity, $\gamma_{0}$ is the spontaneous emission rate of the emitter in free space, Q is the cavity quality factor, $V_{mode}$ is the mode volume, λ is the emission wavelength, and n is the refractive index of the medium. According to Fermi’s golden rule, $\gamma_{sp\left(r\right)=\frac{\pi \omega }{3\hbar \epsilon_{0}}{\left|p\right|}^{2}\rho \left(r,\omega \right)}$, ω is the emission frequency, p is the transition dipole moment of the emitter, r is the position, $\varepsilon_{0}$ is the permittivity of free space, and ρ(r,ω) is the electromagnetic local density of states at frequency ω [72]. A typical plasmonic structure has a great Purcell factor due to the nanoscopic mode volume and the high cavity quality factor. Thus, the luminescence materials in a plasmonic resonant cavity can obtain an obvious acceleration of their spontaneous emission and have a sufficient emission enhancement. Nevertheless, if the excited LSPR releases the energy in a non-radiative decay rate, the non-radiative transition rate would be increased. This can be taken as energy transfer from the luminescent molecules to the metal NPs, which we will discuss in Section 3.3.

Not only spherical particles of different sizes can be used in the enhancement of luminescence, particles with other morphologies can also be applied in this field. Lakowicz and his co-workers have done plenty of research on the luminescence–metal interaction, including lanthanides. They developed core-shell nanocomposites to enhance the luminescence of lanthanides [73]. Choi and his co-worker found that when the emission spectra of Eu^3+^ ions overlap with the LSPR band of Ag NPs, the luminescence intensity would be improved, and they further elucidated the distance dependence of plasmonic-influenced radiative transitions [74]. Mawlud compared the role of Au and Ag NPs on the radiative properties of Sm^3+^ ions, and analyzed the optical properties of Sm^3+^ ions with the help of Judd-Ofelt(J-O) theory. For the increased emission transition rate, it is mainly caused by two reasons. One is the local field effect, and the other is the energy transfer between Ag/Au NPs and Sm^3+^ ions [75]. We developed the Ag nanoprisms [76], nanocubes [77], Ag NPs with different shapes [78], and Ag island films [79] to enhance the radiative decay rate of lanthanide complex. We found that besides spectral overlap, the emission enhancement is also influenced by some other factors, such as excitation wavelength, dopant concentration of lanthanide materials, distribution of NPs, and surrounding environment. For lanthanide materials, the luminescence properties are different under different wavelength excitations. Moreover, the effect of plasmonic NPs on luminescence is also affected by the excitation wavelength. For plasmonic NPs, too-high concentration or too-aggregated nanoparticles will cause quenching. Further discussion about quenching can be found in Section 3.4.

Except the referenced particles, many other shapes or other kinds of particles were used to enhance the luminescence, such as nano-stars, nano-caps, and nano-hexagons [80,81,82]. Among NPs with different shapes, the most promising nanoparticle is metal nanorods (NRs) due to their tunable LSPR band and large-scale production without high cost [83]. These properties mean the NRs have the potential to be an ideal candidate to regulate the luminescence. Liu simultaneously enhanced the excitation and emission efficiency of surrounding luminescent molecules by the transverse LSPR and longitude LSPR modes of gold nanorods (GNRs) [84]. NRs were also applied in the lanthanide materials. Lakowicz and his co-workers reported the optical properties of Eu^3+^ based on Ag NRs, which exhibit two plasmon absorption bands and close to absorption and emission bands of Eu^3+^ chelates, respectively [85]. Then, they further reported the first observed enhancement of luminescence from single lanthanide chelate on Ag NRs. The photoluminescence(PL) enhancement factor was 280-fold, the excitation enhancement was 7-fold, and the emission enhancement was 40-fold [86]. Similar to the above work, Wang provided an efficient heterostructure Au/GdVO_4_: Eu core-shell NRs, and the quantum yield was enhanced as high as 17% [87]. We also prepared Eu^3+^- polymethyl methacrylate(PMMA) shell-covered gold NRs on glass substrates and obtained the enhanced luminescence and tuning lifetime. Fluorescence lifetime can be adjusted by the aspect ratio of gold nanorods and it can be applied to information storage [88].

**Figure 4:**
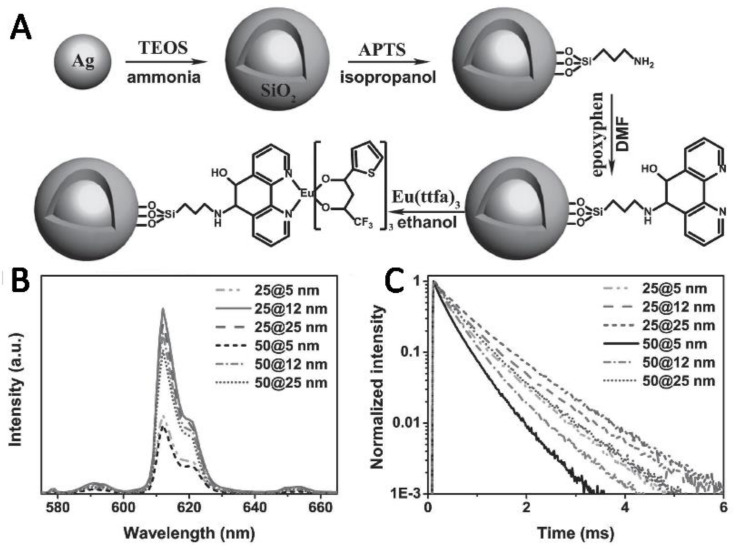
(**A**) The sketch map of Eu chelate grafted onto Ag@SiO_2_ nanoparticles (NPs). (**B**) The co-responding luminescence spectra of samples with a 25 nm Ag core. (**C**) The lifetime of samples. Reference [99].

Core-shell NPs can also be widely applied in the enhancement of luminescence due to the tunable distance between metal NPs and lanthanide complexes. Haldar and Patra investigated the enhancement and quenching of Eu^3+^ emission in the presence of Au-ZnO core-shell NPs and Au NPs. The luminescence of Eu^3+^ was enhanced due to the modulated radiative and nonradiative rate of Eu^3+^ [89]. Lakowicz and his co-workers developed the silica core-silver shell encapsulated with Eu^3+^ complex and found that accompanying with the decrease of lifetime, the emission of Eu^3+^ complex was significantly enhanced by varying the thickness of the metal shell. The mechanism was very complicated, and they offered a simple discussion and attributed the results to the near-field coupling interactions of lanthanide materials with the metal shells. The coupling interaction induced the increase of the radiative decay rate [90]. Zhang et al. prepared and investigated the plasmon-enhanced UC luminescence of NaYF_4_: Yb, Er@SiO_2_@Ag core-shell nanocomposites. They found that the enhancement depended on the competition between energy transfer and enhanced radiative decay rates, which had a great relationship with the separation distance between Ag NPs and UC nanocrystals [91]. Chu et al. demonstrated both the excitation and the emission enhancement of lanthanides by Ag@SiO_2_ NPs [92,93]. They also found that the enhancement of luminescence is sensitive to both the LSPR wavelength of Ag@SiO_2_ NPs and the distance between the plasmonic NPs and the lanthanide complex [94]. Then, they further presented that the luminescence enhancement depended on the size of the Ag@SiO_2_ NPs and the composition of the lanthanide complexes by using nine types of lanthanide complexes and seven kinds of Ag@SiO_2_ NPs. The enhancement factor was higher for the better spectral overlap between the emission bands of lanthanide complex and LSPR absorption bands of Ag@SiO_2_ for the same ligand [65]. Runowski claimed that KY_3_F_10_: Yb^3+^, Tm^3+^@SiO_2_-NH_2_@Au core/shell nanomaterials exhibited tunable UC emission, which can be tuned from bright blue to blue-violet. They mainly attributed the results to the spectrally selective reabsorption in the presence of Au NPs because of the overlap between the plasmonic absorption of Au NPs and the emission bands of Ln^3+^ [95]. Zheng and his co-workers prepared Au@SiO_2_/NaYF_4_: Tb^3+^, Yb^3+^@NaYF_4_ nanocomposites by using the electrostatic effect to combine Au@SiO_2_ NRs with NaYF_4_: Tb^3+^, Yb^3+^@NaYF_4_ NPs. They obtained the plasmon-enhanced UC luminescence and quantum cutting by varying SiO_2_ shell thickness and concentration of Au@SiO_2_ NPs, while the emission spectrum matched with the LSPR of NRs [96]. Kang designed and fabricated a hybrid plasmonic UC nanomaterial which was made up of an Au@SiO_2_ NRs and NaGdF_4_:Yb^3+^, Nd^3+^@NaGdF^4^:Yb^3+^, Er^3+^@NaGdF_4_ core-shell–shell up-conversion nanocrystals (UCNCs). They showed strongly enhanced UC luminescence (up to 20-folds) and flexibly tunable UC colors, and these results were induced by the simultaneous excitation and emission enhancement in the Er^3+^ green emission and only excitation enhancement in the blue and red emissions [97]. Similar to the above work, we also demonstrated the enhanced luminescence of Y_2_Ti_2_O_7_: Er^3+^/Yb^3+^ films when Ag/Au NPs were doped in the films [98]. In order to get satisfactory tunable luminescence, we further synthesized the enhanced luminescence and the tunable lifetime of Eu^3+^ ions by grafting europium chelate onto the Au@SiO_2_ NPs, as shown in Figure 4, and the luminescence was regulated by the thickness of the silica shell. We claimed that Eu-grafted core-shell NPs exhibit a brighter emission and a wider tunable range lifetime, which was sensitive to the separation distance [99]. We recently obtained the 263-fold enhancement and the flexible luminescence colors in Eu^3+^/Tb^3+^ co-doped PMMA films by using the Au@SiO_2_ NRs [100,101,102]. The core-shell structure is not only widely used to enhance luminescence, but is also an effective approach to eliminate quenching.

### 3.3. Quenching

Plasmonic NPs can effectively absorb energy when they are coupled with fluorescent molecules to form fluorescence resonance energy transfer pairs. This strategy is sensitive to spectra overlap and the distance between Luminescent molecules and Metal NPs [103,104,105]. The distance, wavelength, size, structure, and concentration dependences of the plasmonic NPs can also affect luminescence quenching, which can be analyzed by the theories about nonradiative energy transfer between dipoles [105].

As discussed above, the competition between luminescence enhancement and quenching is mainly dependent on the distance between the luminescent molecules and the metal NPs. Since the 1980s, one of the mechanisms for the quenching was that for PL quenching due to energy transfer from the luminescent molecules to metal NPs, the energy transfer efficiency was inversely proportional to the third power of the distance (~d^−3^) [106,107]. Typically, if the distance between them is less than several nanometers, the luminescence is efficiently quenched because of the non-radiative energy transfer [45,100]. As noted in References [108,109], at a small distance, the luminescence would be quenched due to the non-radiative energy transfer from the luminescent molecules to the metal NPs.

The previous section on excitation and emission enhancement included the quenching effects in the study of luminescence enhancement, whereby the luminescence quenching would be obtained when the distance between luminescent molecules and plasmonic NPs is very close [47,48]. Liu reported the quenching induced by the non-radiative energy transfer when the distance was smaller than 55 nm [110]. Afterward, researchers attributed metal NPs-induced quenching mainly to two reasons. One was spectra mismatching between the LSPR band and the emission/excitation spectra of luminescent molecules, while the other was the competition of three processes, including the increased excitation rate due to the local field enhancement, the enhanced radiative decay rate due to the Purcell effect, and quenching due to the non-radiative energy transfer from Ln^3+^ ions to metal NPs. It is remarkable that all the processes above were greatly dependent on the distance between the luminescent material and metal NPs [110,111,112,113].

**Figure 5:**
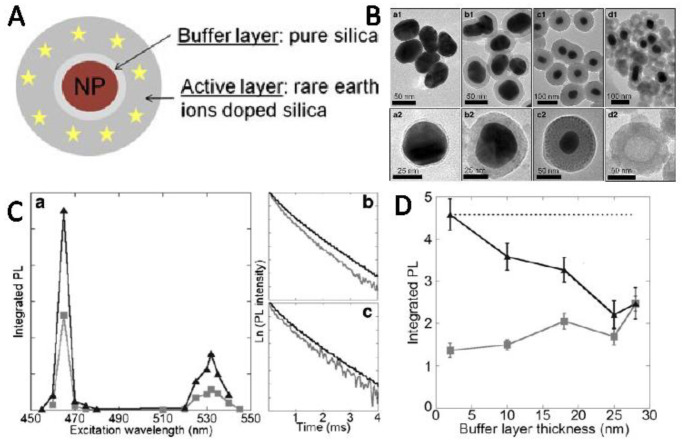
(**A**) Schematic illustrations of multilayer nanostructures. (**B**) Transmission Electron Microscope (TEM) of Au@SiO_2_ nanostructures with different silica shells. (**C**) The excitation spectra when the emission wavelength is 615 nm (**a**), and the decay curves under 465 nm (**b**) and 532 nm (**c**) excitation. (**D**) Integrated PL intensities excited at 532 nm of sample-loaded (gray closed squares) and dissolved (black closed triangle) Au NPs. Reference [117].

The distance of particles and lanthanide ions can be regulated by core-shell particles. There are many reports on the regulation of Ln^3+^ luminescence by core-shell particles. The quenching effects in the previous Section 3.1 and Section 3.2 were included by varying the distance. We will only introduce some of them here. Chu and his co-workers reported that the luminescence of the nanocomposites strongly depended on the SiO_2_ shell thickness, where the luminescent quenching was presented when the shell thickness was very small [65,92,114]. Wu and his co-workers obtained similar results by the non-radiative energy transfer from Au NPs to Eu^3+^ ions, with a distance of 5 nm [115]. Liu et al. synthesized the Au@NaYF_4_: Tb^3+^ core-shell NPs as a luminescence energy transfer system. The distance between Au NPs and Tb^3+^ ions was very close, and meanwhile, the Au NPs absorption matched well with the major emission of Tb^3+^ ions. The luminescence at 489 and 543 nm were both quenched due to energy transfer from Tb^3+^ to Au NPs. They also found a good linear correlation between the emission peak intensities of Tb^3+^ and the Au NPs content. The luminescence quenching is not only obtained at small distances, sometimes, it can also be present at long distances [116]. The coupling between Au NPs and lanthanide ions in the system of Au@SiO_2_@SiO_2_:Eu^3+^ by adjusting the distance between the Eu^3+^ and the Au core is studied by Durupthy and his co-workers. The luminescence quenching was observed for all studied SiO_2_ buffer layer thicknesses, as shown in Figure 5. Strong quenching was experimentally evidenced when the coupling distance of Eu^3+^ ions and plasmonic core was increased to 28 nm. After the systematic study, the researchers attributed the quenching at such long distance to a re-absorption of the emitted luminescence by the gold cores, not to the non-radiative decay rate [117].

Along with the distance dependence and the match of the LSPR band and the emission spectrum, concentration and size of plasmonic NPs can also affect the metal-induced quenching [104]. For lanthanide, Deki et al. reported that the particle concentration depended on the emission intensity. It was deduced that Ag NPs could serve as both an enhancer and a quencher. The luminescence intensity had a great relationship with the size and shape of Ag NPs. In their work, bigger particles showed larger quenching in the lower particle concentration region [118]. Song et al. also found that Ag NPs acted as both enhancers and quenchers for the luminescence of the Eu complex, and the luminescence depended on both Eu complex and Ag NPs concentrations. When concentration of the complex was low (25 μm), the Ag NPs tended to act as quenchers due to the absorption competition of Ag NPs and Eu complex, the reabsorption of Ag NPs to the emission of Eu^3+^, and the energy transfer from Eu^3+^ to Ag NPs. [119]. Marimuthu and his co-worker also reported the luminescence quenching when the Ag concentration was large (>0.5 wt%) due to the reversed energy transfer process from Eu^3+^ ions to Ag NPs [54]. A similar result was obtained by Zhao et al. [56,120]. Gonzalez synthesized Au NPs with different sizes and discussed their influence on the luminescence of UCNPs. The strong luminescence quenching was observed for small AuNPs due to resonance energy transfer, and the reduction of luminescence quenching was observed with the further increase of AuNPs size [121].

Except the factors referred to above, other factors can also influence the quenching deduced by plasmonic NPs for the different materials. Sohn embedded Au NPs in one-dimensional Eu^3+^ and Tb^3+^ hydroxide and oxide nanostructure, and the PL intensity was quenched by dipole–dipole coupling between Au NPs and luminescent centers. The intensity was further quenched by thermal annealing. Jimenez demonstrated the PL quenching and the increase in decay times with the increase in heat treatment holding time, which increases Ag particle volume fractions in the nanocomposites. A similar result was also obtained by Dousi et al. [122,123,124,125].

Except the factors referred to above, the unfavorable composition of metals and lanthanides can also affect the quenching. Eriksen et al. reported that gold NPs might not be suitable for increasing the efficiency of UC at 1523 nm excitation [126]. Simovski and his co-worker claimed that when the nanoantenna is a simple Ag nanoparticle nearby quantum emitter, the fluorescence is suppressed across the entire spectrum. However, if the nanoantenna is composed of two plasmonic NPs with a small space, fluorescence quenching will not occur [127]. For enhancing lanthanide luminescence, quenching is disadvantageous, but the quenching caused by plasmonic NPs is very beneficial in other fields, such as redox reactions [128] and fluorescence detection [129].

### 3.4. Plasmon-Enhanced Luminescence by Modulating FRET

FRET is the process where the donor absorbs the incident light and then transfers the energy to the acceptor in a non-radiative form. If the emission spectrum of the donor and absorption spectrum of the acceptor match well, FRET occurs between the excited donor and acceptor when the distance between them is not much larger than the Forster radius, R. If two fluorescent chromophore groups are close enough, the donor molecule absorbs photons of a certain frequency, and it is excited to a higher electron energy state. Before the electron returns to the ground state, the energy is transferred to the neighboring receptor molecule through dipole interaction. The efficiency of energy transfer is related to the overlap degree of the donor’s emission spectrum and the recipient’s absorption spectrum, the relative orientation of the transition dipole between the donor and recipient, and the distance between the donor and the recipient. FRET is a kind of radiant energy transition through the electric dipole of inter-molecular interactions. The excited donor transfer energy to the excited state of receptors, which will reduce the donor fluorescence intensity and make the receptor launch much fluorescence. The plasmonic NPs can serve as sensitizers and influence the emission of lanthanide ions. The LSPR can enhance FRET between donors and acceptors. It can improve the performance of FRET by increasing the FRET interaction distance, resulting in higher sensitivities and enhanced efficiencies [130]. LSPR can be excited by using the appropriate wavelengths, transferring the energy to the lanthanide ions in a non-radiative form, where the Ln^3+^ ions were excited to their excited state which finally triggered the radiative transition at the emission wavelength.

Many researchers reported an interaction between FRET and metal NPs [131,132,133,134,135]. For lanthanide-doped materials, the FRET is applied to enhance the luminescence of materials by the indirect excitation, because the luminescence efficiency was limited due to the forbidden internal 4f-4f transitions direct excitation with the Ln^3+^ activator. Generally, the energy donor sensitizer might be the ligands in the case of lanthanide complexes or other Ln^3+^ ions in the case of inorganic compounds [136,137]. The plasmonic NPs can also serve as the energy donor. The energy transfer occurs from metal NPs to Ln^3+^ ions, which results in the increased radiative decay rate. Malta et al. studied the effect of metallic NPs on the Eu^3+^ luminescence in glass and glass ceramics. They found that except the enhanced local field in the vicinity of Eu^3+^ ions due to Ag NPs, the energy transfer between Eu^3+^ ions and Ag NPs also affects the luminescence of Eu^3+^ [138]. Eichelbaum distinguished the luminescence energy transfer between small, molecule-like plasmonic NPs and the plasmonic enhancement in soda-lime silicate glass and Ln^3+^ ions. They observed a 250-fold lanthanide luminescence enhancement due to energy transfer from small NPs to Ln^3+^ ions [139]. Rivera and his co-workers presented a significant enhancement of Er^3+^ ions and attributed the enhancement to the formation of the electric dipoles (EDs) in tellurite glass with NPs. The EDs caused the great enhancement of Er transition due to the energy transfer caused by the coupling of NPs dipoles and Er^3+^ transition [140]. Sahar et al. also reported the energy transfer between Ag and Er^3+^ on zinc-tellurite glass [141]. Many researchers found that besides the possible plasmonic enhancement due to NPs, the energy transfer from the NPs to Ln^3+^ ions might also be one of the reasons for the enhanced luminescence intensity [142,143,144].

As well as the energy transfer between plasmonic NPs and Ln^3+^ ions, the plasmonic NPs can also affect the energy transfer between Ln^3+^ ions (or Ln^3+^ ions and other ions). Reil experimentally and numerically showed the strong modification of FRET between Eu^3+^ and CY5 by tuning the LSPR band of nearby plasmonic NPs [15]. Typical energy transfer occurs between Eu^3+^ and Tb^3+^ ions. Some researchers used Ag NPs to the Eu^3+^/Tb^3+^ co-doped materials [145]. We also found the luminescence enhancement of Eu^3+^-Tb^3+^ co-doped system due to the dual enhancement mechanisms of FRET and plasmonic enhancement in the presence of Au@SiO_2_ NRs. The luminescence of binary lanthanide (Eu^3+^/Tb^3+^) complexes was enhanced and modified. The maximum emission enhancement was over 100-fold, and the color of luminescence changed from green to yellow. Meanwhile, we verified the increased energy transfer efficiency from Tb^3+^ to Eu^3+^ [101,102].

More research has been focused on how the plasmon affected the energy transfer on the UC luminescence. Lanthanide-doped UC nanocrystals have been given great attention owing to their unique optical properties and great potential applications in optogenetics [146], nanoscopy [147], and lasers [148]. However, UC nanocrystals also face critical challenges in a variety of practical applications because their absorption cross-section is small and luminescence efficiency is generally low. Enhancing the luminescence efficiency of UC nanocrystals by introducing metal NPs has become a trend due to the unique advantages of metal NPs. Sun presented the enhanced UC luminescence in Ln^3+^-doped nanocrystals by plasmonic structures. They indicated that plasmonic structures not only enhanced the electric field, but also increased the energy transfer rate from Yb^3+^ to Er^3+^ ions by 6-fold [149]. Then, they further used a gold pyramid to tune UC luminescence [150]. Park et al. presented the enhanced luminescence of UC nanocrystals on nanograting in the weak excitation regime, which contributed to the increased absorption and Forster energy transfer [151]. Then, they further reported a systematic study on the increased energy transfer UC process of NaYF_4_: Yb^3+^, Er^3+^ NPs on plasmonic nanograting structure. The internal UC efficiency was enhanced from 36% to 56% by the plasmonic nanograting [152]. Yang et al. improved the UC luminescence of Nd^3+^-sensitized NaYF_4_: Yb^3+^, Er^3+^ NPs by using tunable plasmonic Au films with ultra-broad plasmonic absorption. Besides the excitation field enhancement, the enhanced energy transfer was also obtained [153]. Elrafei and his co-workers used gold NPs to enhance the energy transfer process. For green emission, the efficiency was increased from 1.58% to 13.52%, while it was increased from 0.007% to 0.234% for the red one. General expressions of decay rates, optical field enhancement, and their effects on transitions’ probability were also presented [154].

Table 1 provides an overview of enhancement mechanisms and luminescence enhancement factors of a portion of the investigations.

## 4. New Strategies

Many researchers have proved that the plasmonic NPs can enhance the luminescence efficiency and improve the optical properties of lanthanide materials. However, the reported enhancement so far has a large gap with the theoretical enhancement. The current development of plasmon-enhanced luminescence of lanthanide materials needs to be further improved. In order to make full use of the LSPR effect, many new strategies have been recently proposed. Besides the conventional noble metal NPs, such as Ag and Au NPs, some semiconductor NPs were developed to be used for plasmonic applications [155]. Moreover, the coupling structures which can form the dual LSPR effects based on two different kinds of NPs, or different types of the same kind of NPs or gap-mode nanocavities, were used to enhance luminescence.

Recently, the new constructions of plasmonic NPs on the enhanced luminescence were used. Li and his co-workers realized the plasmon-enhanced luminescence of phosphors by shell-isolated Ag NPs as satellites which were made up of metal cores and silica shells. The luminescence efficiency of samples was enhanced due to the acceleration of radiative rates generated by the satellites [156]. Quan et al. presented plasmonic modulated UC luminescence through the fabrication of two-dimensional binary nanoparticle superlattices via self-assembly of spherical NaREF_4_: Yb^3+^/Er^3+^ UCNPs and Au NPs [12]. Yolk-shell structures have emerged in the last decade or so, first demonstrated by Somorjai et al. [157]. Compared to the core-shell structure, there is a void space between the core and shell material in the yolk-shell nanostructure. Other researchers had used yolk-shell nanostructure to enhance the luminescence of lanthanide [158,159,160], which may be a candidate to improve the luminescence.

Along with the noble metals, such as Ag and Au NPs, the semiconductor plasmon NPs can also exhibit the broad, tunable, and strong LSPR. The LSPR of the semiconductor NPs can be extended to the near-infrared range. The semiconductor NPs not only have tunable LSPR but also have the ordinary characteristics induced by semiconductor materials, such as the nonlinear effect, the two-photon absorption effect, and so on. For instance, in addition to the LSPR effect, Cu_2-x_S NPs have a direct bandgap of ~2.5 eV, which varies with structure and size and would cause a two-photon effect under a high excitation power [161,162,163]. Thus, the semiconductor NPs provide a new approach for improving the optical properties of lanthanide materials. Song et al. reported the obviously enhanced UC luminescence by using Cu_2-x_S NPs, which induced the enhancement of the excitation of UCNPs and the increase of energy transfer from Yb^3+^ to Er^3+^ [161,162]. Then, they further realized UC enhancement based on Cu_2-x_S NPs, and they attributed to the enhancement to the synergistic interaction of the LSPR effect, the nonlinear effect, and the photonic crystal effect [163], and enhanced the power conversion efficiency of solar cells [164]. Their results provide a new strategy for improving luminescent materials and highlight the novel application in photonics. Yang and his co-workers prepared the urchin-like WO_2.72_ which showed the LSPR in the near-infrared region and realized the increase of the UC luminescence due to the WO_2.72_ film-enhanced excitation field [165]. Gao and his co-workers prepared core-shell hybrid structure Cu_1.8_S@NaYF_4_: X%Yb^3+^@NaYF_4_: Yb^3+^, Er^3+^, and they further enhanced the LSPR-coupled UC luminescence by adjusting the intermediate spacer NaYF_4_: X%Yb^3+^ and Yb^3+^ concentration. The suitable NaYF_4_: X%Yb^3+^ with proper thickness and the optimum Yb^3+^ concentration not only avoided luminescence quenching but also excited more photons [166]. Peng and her co-workers synthesized a new core-shell-structured CuS@YF_3_: Eu^3+^ and enhanced the luminescent property induced by the plasmon-enhanced localized electric field effect at the interface of CuS@YF_3_: Eu^3+^ NPs [167]. Many other research works about the plasmon-enhanced luminescence by semiconductor NPs have been presented, such as the results in References [168,169,170,171]. All the results indicated that these semiconductor NPs have remarkable potential for improving the luminescence of lanthanide materials.

Moreover, in recent years, some researchers have reported on the outstanding luminescence enhancement by using the coupling structures, whereby the coupling nanostructure offers a means for tailoring LSPR. Here, we mainly introduced two kinds of coupling nanostructures, one kind is the plasmonic metal/semiconductor hybrid coupling nanostructures, while the other is the coupling of different metal nanostructures, such as the coupling of metal film and metal film, metal NPs and metal film, and so on. The plasmonic metal/semiconductor hybrid coupling nanostructures have become an excellent platform to improve the optical properties, which can combine LSPR together with semiconducting properties and finally achieve positive behaviors. The hybrid nanostructures can also produce new LSPR behaviors which are quite different from those of bare metal NPs. For instance, for the metal-semiconductor Au-Cu_2-x_S, the optical extinction spectra of the dual plasmonic nanostructure show two distinct peaks in visible and near-infrared range, originating from the metal and semiconductor domains, respectively. For metal/semiconductor hybrid nanostructures, the well-designed hybrid nanostructures have been applied to realize luminescence enhancement [172]. Yang and his co-workers obtained strong enhancement of monolayer MoS_2_ PL emission by coupling unique Ag nano-ridges, which formed via the precisely controlled rapid thermal annealing process [173]. Guidelli and his co-workers found that both strongly enhanced UV emission from the ZnO when the samples were excited at 325 nm by using ZnO/Ag and ZnO/Au particles. The emission intensity increased with the metal nanoparticle concentration [174]. Recently, Cong and his co-workers enhanced the overall UC luminescence intensity by Au/W_18_O_49_ heterostructures and found that the enhancement factor can reach to 1108-fold due to the increased excitation and the coupling of LSPR with emission light [175]. Cheng and his co-workers introduced the dual plasmonic Au-CuS heterodimer to NaGd_4_: Yb^3+^/Er^3+^ nanocrystals and enhanced the UC luminescence of nanocrystals. The local enhanced field effect and Purcell effect both contributed jointly to the enhancement. This work provides the possibility to carry out plasmonic luminescence enhancement by metal-semiconductor dual plasmonic antennas [176].

**Figure 6:**
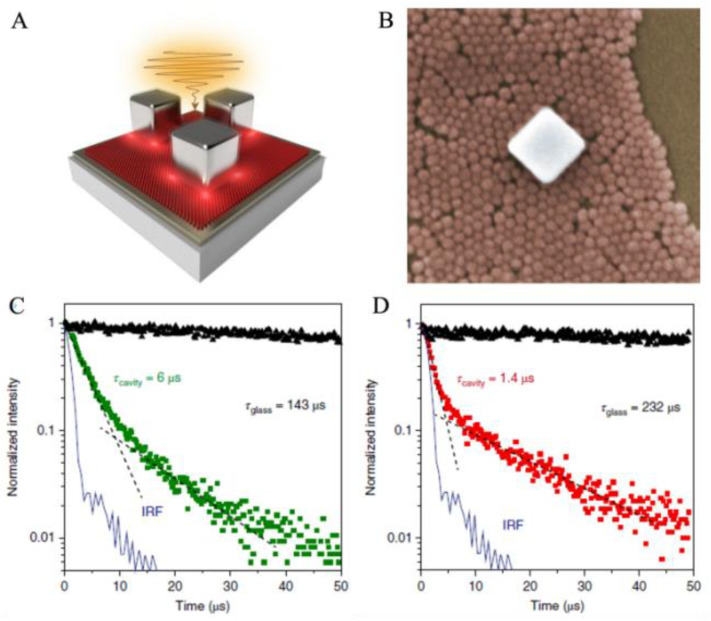
(**A**) Schematic of plasmonic cavity. (**B**) Scanning electron microscopy (SEM) image observed from a typical plasmonic cavity (scale bar, 100 nm). (**C**) Comparison of luminescence decay for up conversation nanoparticles (UCNPs) deposited on a glass slide (black) and on the nanocavity mode (green) for the emission at 554 nm. (**D**) Comparison of the luminescence decay for UCNPs deposited on a glass slide (black) and on the nanocavity mode (red) for emission at 660 nm. Reference [179].

With the development of plasmonic metal/semiconductor hybrid coupling nanostructures, many other coupling systems were designed in recent years. Song et al. effectively improved the luminescent intensity of UCNPs and the luminescent enhancement of CsPbCl_3_ nanocrystals [37,177] by coupling with surface plasmon and photonic crystals. Park and his co-workers prepared metal-insulator-metal (MIM) nanostructures and obtained over 1000-fold enhancement of luminescence by the MIM nanostructures [178]. Liu and his co-workers gained a deeper understanding about Purcell effect-enhanced UC luminescence, which was shown in Figure 6. They regarded the coupling nanostructure of Ag NP and Au film as a plasmonic nanocavity and quantified the luminescence enhancement of UCNPs by the nanocavity. The emission amplification was achieved by four to five orders of magnitude and the spontaneous emission rate was increased 166-fold. The substantial acceleration of spontaneous emission and the significant luminescence enhancement were attributed to the plasmonic nanocavity with a large Purcell factor due to the nanoscopic mode volume [179]. Zhang and his co-workers used nitrogen-doped carbon clots (N-CDs) and Ag nanoprisms to inhibit the fluorescence resonance energy transfer [180]. Sun et al. reported a novel platform based on the reduced graphene/molybdenum disulfide films for plasmon-enhanced fluorescence, which has less optical loss and high sensitivities [181]. Yu et al. improved the photocatalytic performance and degradation rate by using metal-doped ZnO nanofilms grown on graphene-coated flexible substrates [182]. Shao and his co-workers reviewed the two-dimensional (2D) plasmonic nanomaterials in the field of sensing. They reported that researchers had utilized graphene, the metallic NPs/graphene hybrid nanostructures, and metal NPs-coupled metal film systems as the plasmonic nanomaterial for LSPR sensing [183]. All of these coupling systems were usually constituted by hybrid nanostructures, which offered an ideal platform to greatly enhance the luminescence.

The new constructions of plasmonic NPs, hybrid nanostructures, and the coupling systems may be the ideal platform for future modulation of luminescence. These new platforms not only provide a useful tool for investigating the interactions of photon–matter, but also enable various potential applications in plasmon-enhanced luminescence of lanthanide materials.

## 5. Conclusions

In recent years, the application of NPs to enhance lanthanide luminescence has made great progress. Using the LSPR effect of plasmonic nanomaterials to enhance the luminescence of lanthanide elements by designing the structure, size, and shape of NPs has become a research trend. The LSPR effect of plasmonic NPs can influence the luminescence of materials in different aspects, including excitation enhancement induced by enhanced local field, emission enhancement induced by the Purcell effect, and quenching induced by energy transfer from Ln^3+^ to NPs in the nonradiative form. Plasmonic NPs can also be used to modulate FRET between an excited donor and an acceptor. Though great progress has been made, there are still some unsolved shortcomings. In the future studies, seeking for well-designed new constructions of plasmonic NPs, hybrid nanostructures, and coupling systems may be the ideal platform and the future trend to greatly enhance lanthanide luminescence.

## Figures and Tables

**Table 1:** Enhancement mechanisms and luminescence enhancement factors.

Reference	Types of Nanomaterials	Enhancement Mechanism	Enhancement Factor
Polman et al. [41]	silver nanocrystals	excitation enhancement	70-fold
Shao et al. [43]	Ag/Si nanostructure	excitation enhancement	82-fold
Xu et al. [46]	Ag NPs	excitation enhancement	23-fold
Araújo et al. [50]	Ag NPs	excitation enhancement	1.6-fold
Fujii et al. [52]	Ag island films	excitation enhancement	220-fold
Xu et al. [53]	Au–Ag alloy island film	excitation enhancement	180-fold
Zhang et al. [54]	Au film	excitation enhancement	36-fold
Francs et al. [57]	core-shell NPs	excitation enhancement	11-fold
Kennedy et al. [58]	Ag@SiO_2_ core-shell NPs	excitation enhancement	24-fold
Kim et al. [61]	Au and Ag nanoshells	excitation enhancement	20-fold
Karmakar et al. [63]	Au-Ag core-shell NPs	excitation enhancement	2-fold
Chu et al. [65]	Ag@SiO_2_ core-shell NPs	excitation enhancement	21.4-fold
Ghoshal et al. [69]	titania NPs	excitation enhancement	30-fold
Zhang et al. [85]	Metal Nanorods	emission enhancement	240-fold
Zhang et al. [86]	Ag Nanorods	emission enhancement	280-fold
Lakowicz et al. [90]	core-shell NPs	emission enhancement	10-fold
Zhang et al. [91]	core-shell NPs	emission enhancement	14.4-fold
Chu et al. [94]	Ag@SiO_2_ NPs	emission enhancement	10.8-fold
Runowski et al. [95]	Au@SiO_2_ NPs	emission enhancement	2.25-fold
Lei et al. [97]	Au@SiO_2_ nanorods	emission enhancement	20-fold
Wang et al. [100]	Au@SiO_2_ nanorods	emission enhancement	263-fold
Wang et al. [101]	Au@SiO_2_ nanorods	emission enhancement	100-fold
Durupthy et al. [115]	Au NPs @ mesoporous silica	quenching	0.30-fold
Liu et al. [117]	Au@SiO_2_ NPs	quenching	0.67-fold
Song et al. [119]	Ag NPs	quenching	0.1-fold
Zhao et al. [120]	Au co-doped TiO_2_	quenching	0.8-fold
Bradley et al. [133]	Au NPs	FRET	2.03-fold
Sohn et al. [134]	Ag NPs	FRET	63.1-fold
Rademann et al. [139]	Au, Ag NPs	FRET	250-fold
Sahar et al. [141]	Ag NPs	FRET	3-fold
Ghoshal et al. [142]	Au NPs	FRET	4.91-fold
Zhou et al. [143]	Ag NPs	FRET	1.62-fold
Shahi et al. [145]	Ag NPs	FRET	2-fold
Nagpal et al. [149]	Au films	FRET	6-fold
Park et al. [152]	nanograting structure	FRET	4-fold
Yang et al. [153]	Au films	FRET	6-fold

## Data Availability

Data available in a publicly accessible repository.
